# A look inside epitaxial cobalt-on-fluorite nanoparticles with three-dimensional reciprocal space mapping using GIXD, RHEED and GISAXS

**DOI:** 10.1107/S0021889813008777

**Published:** 2013-06-07

**Authors:** S. M. Suturin, V. V. Fedorov, A. M. Korovin, G. A. Valkovskiy, S. G. Konnikov, M. Tabuchi, N. S. Sokolov

**Affiliations:** a Ioffe Physical-Technical Institute of the Russian Academy of Sciences, St Petersburg, Russian Federation; b Synchrotron Radiation Research Center, Nagoya University, Nagoya, Japan

**Keywords:** cobalt-on-fluorite nanoparticles, grazing-incidence X-ray diffraction (GIXD), reflection high-energy electron diffraction (RHEED), grazing-incidence small-angle X-ray scattering (GISAXS), epitaxial growth, three-dimensional reciprocal space mapping

## Abstract

In this work epitaxial growth of cobalt on CaF_2_(111), (110) and (001) surfaces has been extensively studied. It has been shown by atomic force microscopy that at selected growth conditions stand-alone faceted Co nanoparticles are formed on a fluorite surface. Grazing-incidence X-ray diffraction (GIXD) and reflection high-energy electron diffraction (RHEED) studies have revealed that the particles crystallize in the face-centered cubic lattice structure otherwise non-achievable in bulk cobalt under normal conditions. The particles were found to inherit lattice orientation from the underlying CaF_2_ layer. Three-dimensional reciprocal space mapping carried out using X-ray and electron diffraction has revealed that there exist long bright 〈111〉 streaks passing through the cobalt Bragg reflections. These streaks are attributed to stacking faults formed in the crystal lattice of larger islands upon coalescence of independently nucleated smaller islands. Distinguished from the stacking fault streaks, crystal truncation rods perpendicular to the {111} and {001} particle facets have been observed. Finally, grazing-incidence small-angle X-ray scattering (GISAXS) has been applied to decouple the shape-related scattering from that induced by the crystal lattice defects. Particle faceting has been verified by modeling the GISAXS patterns. The work demonstrates the importance of three-dimensional reciprocal space mapping in the study of epitaxial nanoparticles.

## Abstract 2

Three-dimensional reciprocal space mapping by X-ray and electron diffraction [namely grazing-incidence X-ray diffraction (GIXD), reflection high-energy electron diffraction (RHEED) and grazing-incidence small-angle X-ray scattering (GISAXS)] was used to explore the internal structure and shape of differently oriented epitaxial Co/CaF_2_ facetted nanoparticles.

## 1. Introduction

In recent years, intense research efforts have been devoted to the study of heterostructures with magnetically ordered ferro- and antiferromagnetic layers. Particular magnetic effects observed in these systems are of great importance for the design of high-density magnetic storage (Nogués *et al.*, 2005 ▶). One of the possible technologies to produce such systems is epitaxial deposition of ferromagnetic metals (Co, Ni, Fe) on nonmagnetic (CaF_2_) and antiferromagnetic (MnF_2_, NiF_2_) insulating fluoride layers grown on Si. Such heterostructures provide a suitable framework to study properties of the two-dimensional magnetically ordered nanoscale systems (Suturin *et al.*, 2013 ▶).

Nucleation and growth of metals on insulating surfaces have been of interest for a long time. The best known systems of this type are noble metals on alkali halides (Venables *et al.*, 1984 ▶; Robins, 1988 ▶). The much higher surface energy of metals than that of insulators results in the Volmer–Weber growth mode. Hence three-dimensional metal islands nucleate on the surface directly, without forming any wetting layer. Therefore a drastic difference between magnetic properties of metal-on-insulator and metal-on-metal systems is observed (Shiratsuchi *et al.*, 2007 ▶; Scheinfein *et al.*, 1996 ▶). Much less is known about the growth properties of metals on alkaline earth fluorides. Growth of Fe and Co on CaF_2_(111) surfaces *via* a defect-induced nucleation mode with no particular epitaxial relationship to the substrate were reported by Heim *et al.* (1996 ▶). Epitaxial growth of α-Fe(110) on the CaF_2_(111) surface was reported by Mattoso *et al.* (1996 ▶). Later on low-temperature epitaxial growth of Co nanoparticles on the CaF_2_(111) and CaF_2_(110) surfaces was confirmed (Yakovlev *et al.*, 2006 ▶; Pasquali *et al.*, 2006 ▶). Very recently a comprehensive study focused on growth mechanisms of Co nanoparticles on a defect-free CaF_2_(111) surface over a wide range of growth temperatures has been reported (Sokolov *et al.*, 2013 ▶). In particular it has been demonstrated that at 773 K Co grows on CaF_2_(111) predominantly with a face-centered cubic (f.c.c.) lattice. This phase becomes stable above 723 K (Lee *et al.*, 1978 ▶), whereas under normal conditions Co has a hexagonal close-packed (h.c.p.) structure. It must be noted that in strained thin films, nanowires (Dubrovskii *et al.*, 2008 ▶) and nanoneedles (Moewe *et al.*, 2008 ▶), metastable crystal phases are observed quite often, *e.g.* an f.c.c. metastable phase has been discovered in Co films on Pt(111) (Ferrer *et al.*, 1997 ▶), Au(233) (Baudot *et al.*, 2004 ▶) and NiO(111) (Mocuta *et al.*, 1998 ▶) as well as in Co nanoparticles on Cu(110) (Gu *et al.*, 1999 ▶). A strained body-centered cubic phase has been revealed in the Co layers on Pt(001) and in a number of other systems (Valvidares *et al.*, 2004 ▶). Given a variety of possible crystal structures, it is important to know which one is realized in a particular system.

The present study focuses on the structural properties of well shaped high-crystalline-quality Co nanoparticles epitaxically grown on (111), (110) and (001) CaF_2_ surfaces by an improved double-stage technique. Control of the cobalt crystallographic orientation through choice of various CaF_2_ faces allowed a detailed all-round view of the nanoparticles from outside (size, shape and orientation) and from inside (epitaxial relations, crystal and defect structure) to be obtained. The structural study carried out was empowered by using modern techniques of three-dimensional reciprocal space mapping, namely electron and X-ray diffraction as well as grazing-incidence small-angle X-ray scattering (GISAXS).

## 2. Experimental

In this work, Co/CaF_2_/Si heterostructures were grown by molecular beam epitaxy. Low miscut silicon wafers of (111) and (001) orientations were cleaned by Shiraki chemical treatment and flash-annealed in ultra-high vacuum conditions. Growth of the CaF_2_ buffer layer was performed from an effusion cell at a deposition rate of 2–3 nm min^−1^. Cobalt was deposited from an e-beam source with the flux kept at 0.2–0.3 nm min^−1^. Co exposures ranged between 30 and 45 nm. Here and below we denote Co exposure as the amount of cobalt sent to the surface. The actual amount of remaining material may be less owing to reevaporation (Sokolov *et al.*, 2013 ▶). It must be noted that for the aims of this work a precise control over the Co exposure is not critical because the shape and internal structure of the nanoparticles is not drastically dependent on exposure (Sokolov *et al.*, 2013 ▶). Reflection high-energy electron diffraction (RHEED) studies have been carried out with an electron energy of 15 keV in a geometry that allowed the incident angle to be changed by rocking the sample. RHEED images were taken with a CCD camera. Sample topography was measured using an NT-MDT atomic force microscope operating at ambient conditions. Grazing-incidence X-ray diffraction (GIXD) and GISAXS were measured at the BL3A beamline at the Photon Factory (PF) synchrotron facility in Tsukuba (Japan). The measurements were made with a photon energy of 12 keV on a four-circle diffractometer with either a point or a two-dimensional CCD detector.

## 3. CaF_2_(111), (110) and (001) buffer layers

To prevent chemical reaction between cobalt and silicon at elevated substrate temperature a CaF_2_ buffer layer was used. CaF_2_ is a widely used chemically inert material suitable for epitaxial growth on Si (Olmstead, 1999 ▶; Vexler *et al.*, 2009 ▶; Sokolov *et al.*, 2004 ▶). From our previous studies (Sokolov *et al.*, 2013 ▶), it follows that Co does not wet the CaF_2_ surface and forms nanoparticles on it. As a result of the weak Co–CaF_2_ interaction most of the nanoparticle properties are not influenced by the substrate. The surprising exception is the Co lattice orientation which, as will be shown below, is unambiguously defined by the orientation of the fluorite surface. Depositing Co on different CaF_2_ faces is the way to look at virtually the same object from different viewing angles as the oriented particles turn different sides to the viewer. This is especially helpful for microscopy and grazing-incidence diffraction studies where the view from the back hemisphere is shadowed by the substrate. In this work, CaF_2_(111), (110) and (001) layers were used to force particular orientations of cobalt nanoparticles.

**Figure 1:**
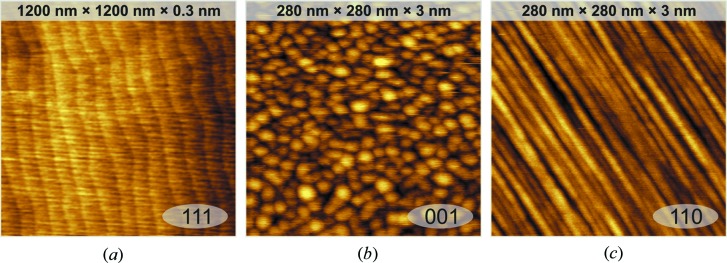
AFM images of (*a*) CaF_2_(111), (*b*) CaF_2_(001) and (*c*) CaF_2_(110) layers on Si.

Typical surface morphologies of the three CaF_2_ surfaces measured by atomic force microscopy (AFM) are shown in Fig. 1 ▶. The CaF_2_(111) layer is grown on an Si(111) substrate following the recipe known to result in uniform films with particularly regular surface morphology (Sokolov *et al.*, 2013 ▶). The first few monolayers are deposited at 523 K and the rest of the fluorite layer is grown at 1023–1073 K. The roughness of such a surface is mainly determined by the silicon surface steps (Fig. 1 ▶ *a*). The CaF_2_(001) surface is prepared by depositing CaF_2_ on an Si(001) substrate at 573 K. At this temperature the orientation of the fluorite layer is the same as that of the Si substrate (Pasquali *et al.*, 2001 ▶). This surface has typical roughness below 1.5 nm and consists of nanometre-sized square pyramidal huts with {111} slopes (Fig. 1 ▶ *b*). To produce a CaF_2_(110) surface with a reduced roughness a modification of the conventional growth procedure (Pasquali *et al.*, 2005 ▶) was implemented. The first CaF_2_ monolayer was grown at 1023–1073 K to produce the interface reacted layer. The rest of the fluorite was deposited at 673 K to produce a microridged surface with ridges of nanometre size having {111} slopes (Fig. 1 ▶ *c*).

## 4. Cobalt nanoparticles: atomic force microscopy

**Figure 2:**
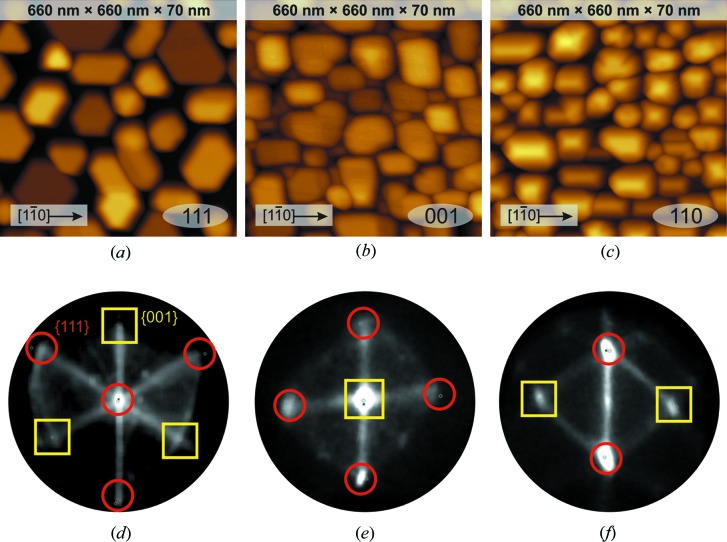
AFM images and the corresponding slope distribution charts of Co nanoparticles on (*a*), (*d*) CaF_2_(111), (*b*), (*e*) CaF_2_(001) and (*c*), (*f*) CaF_2_(110) surfaces.

A variety of growth modes have been investigated in search of optimal conditions suitable for formation of regular arrays of epitaxial Co nanoparticles on CaF_2_(111), (110) and (001) surfaces. The best growth conditions were achieved when a 0.1 nm seeding layer of cobalt was deposited on CaF_2_ at 373 K, while the main Co layer was grown afterwards at 873 K. The AFM images in Fig. 2 ▶ show the results of deposition of 30–45 nm of Co onto the three CaF_2_ surfaces. In all the cases cobalt forms stand-alone islands with width and height defined by the cobalt exposure. The islands look facetted and have well defined shapes: hexagonal on CaF_2_(111), square on CaF_2_(001) and rectangular on CaF_2_(110) surfaces. All of the islands are oriented in the same way, indicating that they are in register with the underlying CaF_2_ layer.

To identify the crystallographic orientation of the island facets, slope analysis has been applied to the AFM images. The slope analysis chart is a stereographic projection plot of the surface normal distribution function showing peaks that correspond to flat regions on the AFM topography map. The charts presented in Figs. 2(*d*), 2(*e*) and 2(*f*) show clear maxima that after fitting can be attributed to slopes with 〈111〉 and 〈001〉 surface normals. It may be deduced that the Co/CaF_2_(111) particles have flat (111) tops, with three {111} and three {001} side facets. The Co/CaF_2_(001) particles have flat (001) tops and four {111} side facets. Finally the Co/CaF_2_(110) particles have a ridge shape with two {111} side facets and two {001} end facets. The fit results allow the conclusion (with reasonable accuracy) that Co islands have an f.c.c. lattice that is oriented identically to the lattice of the underlying CaF_2_ layer.

## 5. Cobalt nanoparticles: electron diffraction

**Figure 3:**
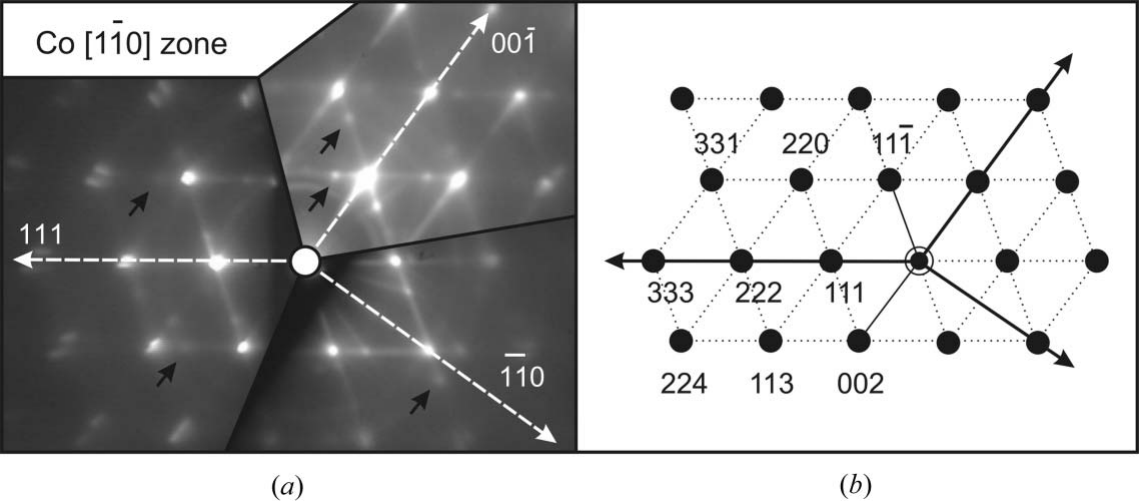
(*a*) The 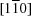-zone RHEED patterns from Co/CaF_2_(111), Co/CaF_2_(110) and Co/CaF_2_(001) samples. (*b*) The patterns are rotated around the zone axis for easier comparison with the simulation.

To get an insight into Co crystal structure and to determine epitaxial relations, RHEED patterns of Co particles on CaF_2_(111), (110) and (001) surfaces have been studied. These patterns taken with the e-beam parallel to the CaF_2_ 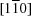 axis are shown in Fig. 3 ▶(*a*). To facilitate interpretation, the images are rotated around the zone axis as if cobalt was grown on the {111}, {110} and {001} faces of a single CaF_2_ crystal. By combining diffraction patterns from samples with different CaF_2_ surface orientation, information from hardly accessible reciprocal space regions below and near the shadow edge are obtained. Fig. 3 ▶(*b*) shows the simulated pattern of the f.c.c. Co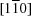 zone. This pattern is perfectly coincident with the experimental ones, which means that in all three cases the f.c.c. lattice of Co is cooriented with that of CaF_2_.

Interestingly, bright 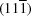 and (111) streaks are present in the observed patterns. The streaks are probably due to the small penetration depth of the incident electrons arriving at grazing incidence to the 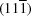 and (111) island facets. For the Co/CaF_2_(001) sample a bright [001] streak is also observed in agreement with AFM slope analysis claiming the presence of flat island tops. The three-in-one representation in Fig. 3 ▶(*a*) is helpful for recognizing that the same regular pattern of additional reflections (a few are indicated by arrows) is present in all of the samples. This pattern would be formed if the dominant lattice is rotated by 180° around the [111] and 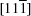 directions. Two other sets of reflections (lying outside the imaged 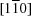 zone) obtained through rotations around the 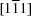 and 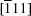 directions are also expected owing to symmetry. In total there exist five lattices – one dominant lattice cooriented with CaF_2_ and four minor {111} twins.

**Figure 4:**
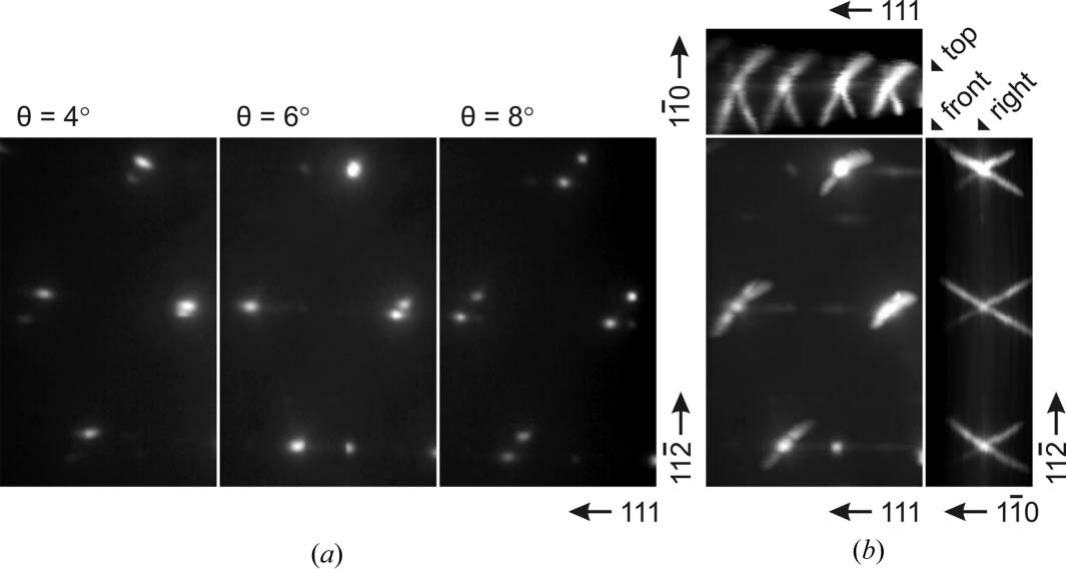
(*a*) The 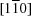-zone RHEED patterns for the Co/CaF_2_(111) sample: Bragg reflection splitting is observed by changing the incident angle. (*b*) Reconstructed projections of the three-dimensional intensity distribution obtained from raw data.

Bragg reflection splitting is the other prominent feature that is present in the RHEED patterns in Fig. 3 ▶(*a*). The splitting occurs as the sample is rocked around the Bragg angle for a given reflection so that the Ewald sphere passes through the corresponding reciprocal lattice node. Fig. 4 ▶(*a*) shows a fragment of the 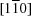-zone RHEED pattern measured for the Co/CaF_2_(111) sample as a function of incident angle. As the Ewald sphere passes through a reciprocal lattice node the corresponding reflection gets divided into two spots that move away slowly decreasing in intensity. The observed behavior means that lines of nonzero intensity are passing through reciprocal lattice nodes. What the orientation and shape of these lines are is not clear unless a special processing is applied to the RHEED patterns. A dedicated processing software has been developed in this work to reconstruct the three-dimensional intensity distribution in the vicinity of the imaged zone. The idea of the method is to process a series of RHEED images taken at gradually increasing incident angle (from 0 to 12° with 0.05° step). After accurate calibration the images (which are essentially spherical cross sections of the reciprocal space by the Ewald sphere) can be stacked together to describe the three-dimensional intensity distribution in front of and behind the imaged zone plane.

Three orthogonal projections can be calculated to visualize the reconstructed three-dimensional intensity distribution (Fig. 4 ▶ *b*). The front projection made along the zone axis is (to a first approximation) a sum of all the patterns in the series. In contrast to the single pattern it shows the complete structure of the zone plane with the superimposed traces of any intersecting objects. In the present case the traces of streaks responsible for reflection splitting are seen as straight lines parallel to the Co[001] direction [Fig. 4 ▶(*b*), front projection]. To understand streak shape and orientation the other two projections are needed. The top projection is equivalent to the pattern that would be obtained if the sample is rotated by 90° around the surface normal. Building this projection can partially replace physical rotation of the sample in the case when it is not available on the sample manipulator. The most nontrivial right projection shows the view of the reciprocal space along the surface normal. This projection is rather like the one usually observed in low-energy electron diffraction.

The three RHEED projections obtained from the Co/CaF_2_(111) nanoparticles are presented in Fig. 4 ▶(*b*). X-shaped Bragg reflections (with 120 and 44° between the X arms) are recognized on the right and top projections. It may be concluded that the X shape is due to Co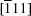 and Co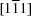 streaks passing through Co reciprocal lattice nodes. Unlike the previously described [111] and 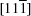 streaks the newly discovered streaks cannot be explained by low penetration depth because the e-beam is not grazing to the corresponding facets.

Crystal truncation rods (CTRs) are the other possible source of streaking (Pietsch *et al.*, 2004 ▶). They appear in the Fourier transform of any object that is truncated by flat borders. If Co islands are indeed facetted as predicted by AFM measurements, the corresponding CTRs would emerge perpendicular to the facets. The truncation rods are propagated beyond the size-defined reflection width and fade fast with wavevector **q** as **q** ^−*N*^. The exponent *N* is equal to two for parallelepipeds, prisms and pyramids and increases gradually to four as the number of facets is increased. At the distance where the CTR becomes separated from the reflection core, its intensity is several orders of magnitude lower than that of the core. However, the streaks observed by RHEED are visible on the same linear brightness scale as the parent reflection and thus cannot be related to facets. Another possible reason of streaking is the variation of lattice constant (Kovats *et al.*, 2000 ▶). The corresponding strain streaks, however, are too short because the variation of the lattice constant can hardly exceed a few percent. Finally, and most likely, streaks may be due to the presence of planar defects perpendicular to the streak direction, in particular, stacking faults in the close-packed structure (Ferrer *et al.*, 1997 ▶). The length of such a streak is defined by the correlation distance in the faulted structure. It must be noted that absolutely the same type of streaks as in the Co(111) islands were recognized in the electron diffraction patterns of the Co(110) and Co(001) islands. The next section will address the streak issue in more detail.

## 6. Cobalt nanoparticles: grazing-incidence X-ray diffraction

Compared with electron diffraction, X-ray diffraction offers higher precision, easier access to reciprocal space and relatively simpler kinematical interpretation. Our X-ray diffraction studies in the first place confirmed that the crystal lattice of Co islands grown on CaF_2_(111), (001) and (110) surfaces is predominantly f.c.c. with a bulk-like lattice parameter of *a* = 3.544 Å and is oriented identically to the lattice of the underlying CaF_2_. An important task of the XRD study was to investigate the nature of the streaks passing through Co reflections by three-dimensional reciprocal space mapping. A series of equidistant cross sections close to Co reciprocal lattice nodes was measured with a two-dimensional detector. To increase surface sensitivity the incident angle was kept constant at 5°. This condition automatically fixed the orientation of the sampling sheet – the reciprocal space region imaged with a single two-dimensional snapshot. To effectively map a parallelepiped region a series of images was taken while moving the sampling sheet in reciprocal space perpendicular to the sheet plane (*i.e.* along the diffracted beam).

**Figure 5:**
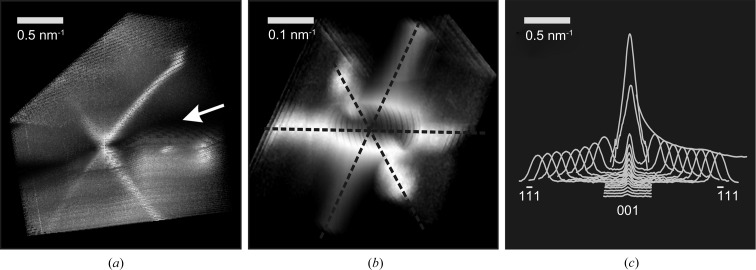
GIXD intensity distribution maps showing streaks around the Co{111} off-specular reflection of (*a*) Co/CaF_2_(111), (*b*) Co/CaF_2_(110) and (*c*) Co/CaF_2_(001) samples. Indicated on the maps is the sample to detector (two-dimensional) distance.

Fig. 5 ▶(*a*) shows a large-angular-size three-dimensional map around the Co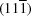 reflection of the Co/CaF_2_(111) sample with clearly visible equidistant sampling sheets. [111] and 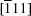 streaks intersecting the 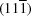 node are present. Light exposure was adapted individually for each image in order to fit into the dynamic range of the detector. A drawback of this technique is that the sampling sheets containing a bright reflection core are taken with low exposure and therefore are insensitive to low-intensity features such as streaks. For example the black gap in Fig. 5 ▶(*a*) (shown by arrow) might contain the hardly visible {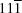} and {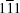} streaks. This problem has been solved by placing a circular beam stopper to mask the bright reflection core. Fig. 5 ▶(*b*) shows a small-angular-size three-dimensional map around the Co(111) reflection recorded in this way for Co/CaF_2_(110) islands. Clearly visible are 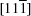, 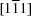 and 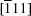 streaks. Another approach of measuring intensity maps was used in the study of Co/CaF_2_(001) islands. Once it was known where to look for the streaks, the corresponding profiles could be measured with a point detector. Fig. 5 ▶(*c*) presents a two-dimensional map of the Co[110] zone in the vicinity of the Co(111) reflection. The map clearly shows the 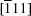 and 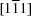 streaks with almost flat (in log scale) intensity profiles.

**Figure 6:**
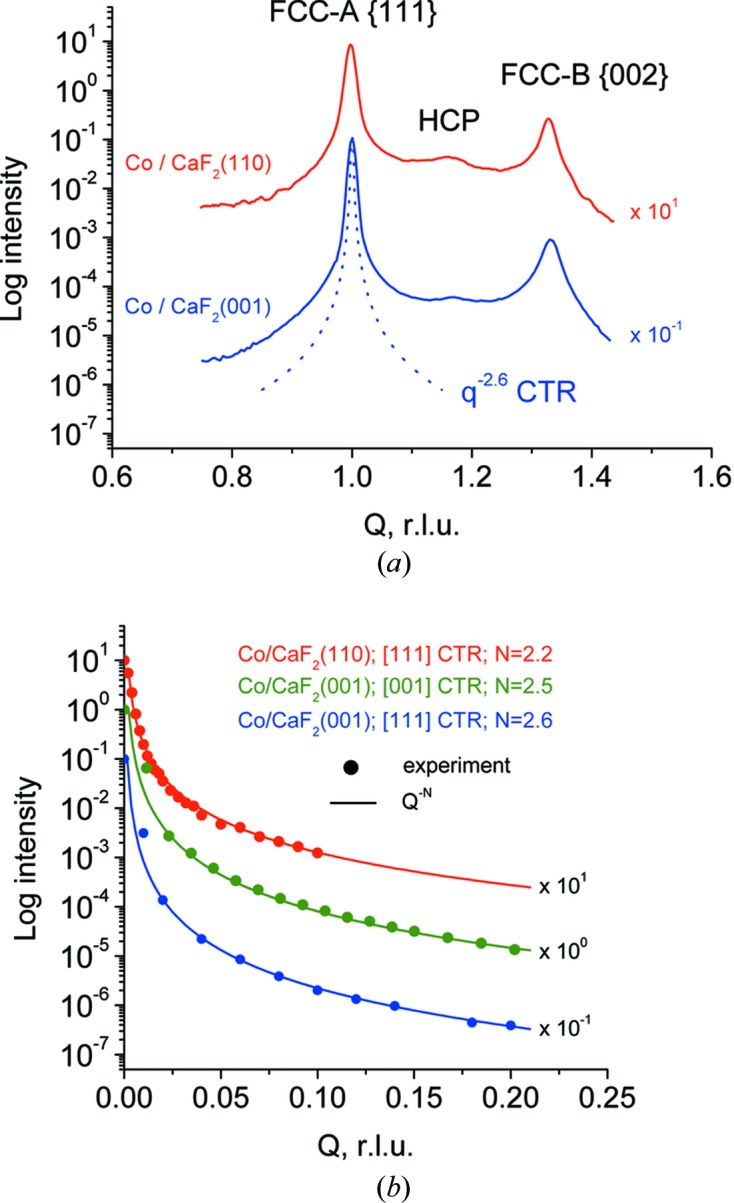
Profiles of (*a*) stacking fault streaks and (*b*) crystal truncation rods passing through off-specular Co{111} reflections. The profiles are shifted along vertical axis for better visibility.

Fig. 6 ▶ shows streak profiles with background subtracted for Co/CaF_2_(110) and Co/CaF_2_(001) samples. A very similar profile (not shown) was measured in the Co/CaF_2_(111) sample. Non-radial (sensitive to the stacking order) 〈111〉 streaks passing through Co{111} off-specular reflections are presented. The dominating features in these profiles are the peak coming from the f.c.c.-A lattice (cooriented with CaF_2_) and a peak belonging to the twinned f.c.c.-B lattice (180° rotation around the 〈111〉 axis parallel to the scan direction). Additionally a minor h.c.p. peak exists in between the two f.c.c. ones.

The profiles indicate that three types of stacking order exist in the scan direction with an f.c.c.-A:f.c.c.-B:h.c.p. intensity ratio of approximately 1000:(10–30):(1–5). From the point of view of a single-domain growth the situation is much improved compared with the growth procedure reported in our previous work (Sokolov *et al.*, 2013 ▶), where Co growth was performed at 773 K, resulting in an f.c.c.-A:f.c.c.-B:h.c.p. ratio of approximately 3:2:1. One reason for the pronounced domination of the single f.c.c. phase in the 873 K samples is that the selected growth temperature is considerably above the h.c.p.–f.c.c. bulk transition.

It was demonstrated by Sokolov *et al.* (2013 ▶) for the sample with cobalt grown at 773 K that it is impossible to fit streak profiles with an incoherent weighted sum of the three different stacking orders. A reasonable fit is obtained only if a few regions with different stacking orders within a single island are assumed to scatter coherently. Stacking faults can appear (i) during ripening of the stand-alone nuclei and (ii) during the coalescence stage. In the first case the stacking order may accidentally change simply because near the h.c.p.–f.c.c. transition temperature the energies of sequences with f.c.c.-like and h.c.p.-like local orders are not drastically different. In the second case the faults appear during coalescence of the phase-shifted islands. Interestingly, the CaF_2_ surface seems to impose an enhanced ordering on Co growth as a result of the 2:3 ratio between the lattice constants of CaF_2_ and Co. This ratio limits the possible phase shifts between adjacent islands to three possible values: 0, 2π/3 or 4π/3. This is opposed to the random phase nucleation in the case when there is no simple ratio between the lattice constants (Ferrer *et al.*, 1997 ▶). In the random phase nucleation case the island fragments grown from different nuclei remain incoherent upon coalescence. In the Co/CaF_2_ case these fragments stay coherent though with stacking faults in between them. As the cobalt growth temperature is increased above the f.c.c.–h.c.p. transition, the stacking faults leading to the appearance of f.c.c.-B and h.c.p. inclusions seem to become suppressed. Inevitably remaining because of the coalescence are the phase-shift faults with f.c.c.-A stacking sequence on both sides of the fault. The liquid phase coalescence mechanism of the Co islands taking place at high growth temperature (Sokolov *et al.*, 2013 ▶) must, to a certain extent, facilitate recrystallization of the islands and reduce the number of stacking faults.

Another challenging task for X-ray diffraction is to detect the 〈111〉 and 〈001〉 truncation rods passing through Co reciprocal lattice nodes. If found these would serve as a convincing proof of island faceting. However the task of detecting 〈111〉 CTRs is not straightforward because of the stacking fault streaks. The latter occupy the same position in reciprocal space and are much brighter because of the slower decay rate. Fortunately stacking fault streaks do not exist along radial directions because the in-plane structure of a crystal plane does not influence the periodicity perpendicular to this plane. Owing to this circumstance the only possibility to detect the [111] CTR is to look at the Co(111) reflection. Fig. 6 ▶(*b*) shows integrated intensity profiles along 〈111〉 and 〈001〉 CTRs passing through Co(111). The 〈111〉 CTR in the Co/CaF_2_(110) sample is well simulated by **q** ^−2.2^ decay in correspondence to what is expected from a side facet of a prism. The absence of a long [111] streak in this position [also evident from the three-dimensional map in Fig. 5 ▶(*b*)] is another proof of the stacking fault origin of the long streaks. The 〈111〉 CTR in the Co/CaF_2_(001) sample shows a **q** ^−2.6^ decay. Similar **q** ^−2.5^ behavior was found for the same sample along the [001] CTR perpendicular to the flat tops of the islands (Figs. 5*c* and 6*b*). Faster CTR decay in Co/CaF_2_(001) samples is probably related to the higher number of facets in the truncated pyramid. For an illustration of how much the CTRs are shorter than the stacking fault streaks, the dashed line in Fig. 6 ▶(*a*) shows the **q** ^−2.6^ CTR-like profile. The presence of truncation rods with a **q** ^−*N*^ decay rate is a fair indication of the particle faceting. However it would be more convincing if independent information on the average particle shape could be obtained. The GISAXS method discussed in the next section is an appropriate technique to decouple the shape issues from the crystal structure.

## 7. Cobalt nanoparticles: GISAXS

**Figure 7:**
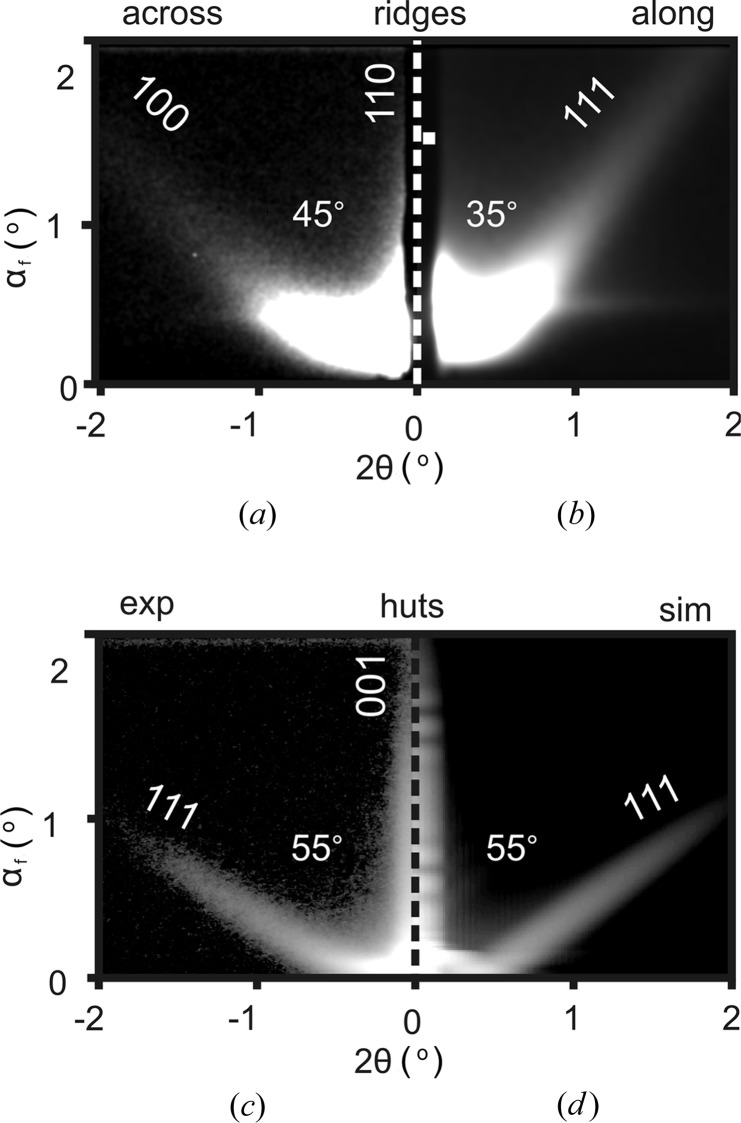
Experimental GISAXS half-patterns from the Co/CaF_2_(110) sample measured (*a*) across and (*b*) along the ridges. (*c*) Experimental and (*d*) simulated GISAXS half-patterns from the Co/CaF_2_(001) sample.

GISAXS was applied to study properties of Co/CaF_2_ nanoparticles such as their shape, size and spatial arrangement. Streaks in the GISAXS patterns were looked for while continuously rotating the sample around the substrate normal. For the Co/CaF_2_(110) sample the streaks appear when the beam travels either across (Fig. 7 ▶ *a*) or along (Fig. 7 ▶ *b*) the CaF_2_ microridges. In the first case the streaks are 45° off-normal and are perpendicular to the expected {100} facets at the island ends. In the second case the streaks appear ∼35° off-normal and correspond to the {111} side facets. These observations are in good agreement with the AFM slope analysis results presented in §4. It is important to note that the observed streaks are not originating from CaF_2_ microridges as was proved by measuring GISAXS from the microridges alone (to be presented elsewhere). Fig. 7 ▶(*c*) shows a GISAXS pattern from Co/CaF_2_(001) nanoparticles. This pattern with two distinct streaks appeared when the beam was parallel to the Co〈110〉 direction. The streaks are ∼55° off-normal and can be identified as perpendicular to the Co{111} planes. A bright specular streak is also present, suggesting that flat regions exist parallel to the substrate surface. Combining GISAXS and AFM data one can conclude that Co islands have the shape of a truncated square pyramid. This shape was used to simulate GISAXS patterns using the ‘simulation annealing’ algorithm implemented in the *IsGISAXS* software (Renaud *et al.*, 2009 ▶). A reasonable fit was achieved for an island having a radius of *R* ≃ 50 (8) nm and a height of *H* ≃ 30 (6) nm. The GISAXS simulation obtained with these parameters is shown in Fig. 7 ▶(*d*). The geometrical parameters determined from GISAXS data are in good agreement with the AFM results. Moreover they are more reliable in evaluating the island aspect ratio.

## 8. Concluding remarks

The results obtained during investigation of cobalt nanoparticle growth on three differently oriented CaF_2_ surfaces may be summarized as follows. Upon low-temperature seeding followed by 873 K deposition, Co grows on the CaF_2_ surface with an f.c.c. structure. The orientation of the cobalt lattice tends to mimic the orientation of the underlying CaF_2_ lattice. A possible reason for these well defined epitaxial relations between materials with such differently sized unit cells is the 3:2 relation between the Co and CaF_2_ lattice constants. A small fraction of cobalt (below 3%) grows with f.c.c. lattices twinned with respect to the {111} planes. An even smaller fraction of the h.c.p. phase (below 1%) is also present. The choice of growth temperature far above the h.c.p.–f.c.c. transition temperature favors growth of a single-domain f.c.c.-A phase.

The growth scenario at the interface between dissimilar materials is usually guided by the surface energy issues. Choosing CaF_2_ surfaces of different orientation could in principle induce variation in cobalt growth because the surface energies of the fluorite surfaces differ considerably. However in all three cases, Co atoms actually see the CaF_2_(111) surface on arrival because the CaF_2_(111) substrate layers exhibit (111) atomically flat terraces while the (110) and (001) surfaces obtain facetted morphology with {111} facets. Therefore Co nucleation at the seeding stage always occurs locally on the CaF_2_(111) surface. This explains the similar Co growth mechanisms observed on the three CaF_2_ surfaces. As a result of non-regular nucleation, there is a high probability that the neighboring particles are nucleated not in phase with each other. Hence stacking faults are formed, as shown by the appearance of long streaks in the electron and X-ray diffraction patterns of the studied samples. Observation of these streaks with RHEED is a good illustration of the power of the three-dimensional approach, which is capable of reconstructing features that lie outside the imaged zone and therefore cannot be observed in a single pattern.

The cobalt nanoparticles are facetted with {111} and {001} planes as follows from AFM direct space and XRD/GISAXS reciprocal space studies. It is important to distinguish the facet truncation rods from the stacking fault streaks. Different decay rates are characteristic for these. The stacking fault streaks decay slowly and do not fall to zero between the f.c.c.-A and f.c.c.-B reflections. In contrast, the truncation rods decay as **q** ^−*N*^, where *N* ranges from 2.2 to 2.6 for the studied samples. To explore the truncation rods separately either radial XRD profiles or GISAXS patterns insensitive to the stacking faults should be studied. The facet information may also be roughly extracted from AFM images by carrying out slope analysis. The AFM topography images are very well complemented by GISAXS data, which represent a statistical average over a large sample area and prove especially useful for describing faceting and the *H*/*R* ratio.

This work in general emphasizes the importance of three-dimensional reciprocal space mapping methods in the study of epitaxial nanoparticles, particularly addressing the very little known approach of carrying out such mapping *in situ* using high-energy electron diffraction.
